# Exome-Wide Search for Genes Associated With Central Nervous System Inflammatory Demyelinating Diseases Following CHIKV Infection: The Tip of the Iceberg

**DOI:** 10.3389/fgene.2021.639364

**Published:** 2021-03-17

**Authors:** Soniza Vieira Alves-Leon, Cristina dos Santos Ferreira, Alice Laschuk Herlinger, Fabricia Lima Fontes-Dantas, Fernanda Cristina Rueda-Lopes, Ronaldo da Silva Francisco, João Paulo da Costa Gonçalves, Amanda Dutra de Araújo, Cláudia Cecília da Silva Rêgo, Luiza Mendonça Higa, Alexandra Lehmkuhl Gerber, Ana Paula de Campos Guimarães, Mariane Talon de Menezes, Marcelo Calado de Paula Tôrres, Richard Araújo Maia, Bruno Miceli Gonzalez Nogueira, Laise Carolina França, Marcos Martins da Silva, Christian Naurath, Aline Saraiva da Silva Correia, Claudia Cristina Ferreira Vasconcelos, Amilcar Tanuri, Orlando Costa Ferreira, Cynthia Chester Cardoso, Renato Santana Aguiar, Ana Tereza Ribeiro de Vasconcelos

**Affiliations:** ^1^Translational Neuroscience Laboratory, Rio de Janeiro State Federal University, Rio de Janeiro, Brazil; ^2^Department of Neurology/Reference and Research Center for Multiple Sclerosis and Other Central Nervous System Idiopathic Demyelinating Inflammatory Diseases, Clementino Fraga Filho University Hospital, Federal University of Rio de Janeiro, Rio de Janeiro, Brazil; ^3^Bioinformatics Laboratory, National Laboratory for Scientific Computing, Petrópolis, Brazil; ^4^Molecular Virology Laboratory, Federal University of Rio de Janeiro, Rio de Janeiro, Brazil; ^5^Department of Radiology, Antônio Pedro University Hospital, Fluminense Federal University, Niterói, Brazil; ^6^Department of Clinical Medicine, Clementino Fraga Filho University Hospital, Federal University of Rio de Janeiro, Rio de Janeiro, Brazil; ^7^Federal Hospital Cardoso Fontes, Ministry of Health, Rio de Janeiro, Brazil; ^8^Department of Neurology, University Hospital Gafrée e Guinle, Rio de Janeiro State Federal University, Rio de Janeiro, Brazil; ^9^Department of Genetics, Ecology and Evolution, Institute of Biological Sciences, Federal University of Minas Gerais, Belo Horizonte, Brazil

**Keywords:** whole exome sequence, chikungunya, pathogenic variants, HLA alleles, inflammatory demyelinating diseases, neurological manifestations in viral infection, acute disseminated encephalomyelitis, myelitis

## Abstract

Chikungunya virus (CHIKV) is a re-emergent arbovirus that causes a disease characterized primarily by fever, rash and severe persistent polyarthralgia, although <1% of cases develop severe neurological manifestations such as inflammatory demyelinating diseases (IDD) of the central nervous system (CNS) like acute disseminated encephalomyelitis (ADEM) and extensive transverse myelitis. Genetic factors associated with host response and disease severity are still poorly understood. In this study, we performed whole-exome sequencing (WES) to identify HLA alleles, genes and cellular pathways associated with CNS IDD clinical phenotype outcomes following CHIKV infection. The cohort includes 345 patients of which 160 were confirmed for CHIKV. Six cases presented neurological manifestation mimetizing CNS IDD. WES data analysis was performed for 12 patients, including the CNS IDD cases and 6 CHIKV patients without any neurological manifestation. We identified 29 candidate genes harboring rare, pathogenic, or probably pathogenic variants in all exomes analyzed. HLA alleles were also determined and patients who developed CNS IDD shared a common signature with diseases such as Multiple sclerosis (MS) and Neuromyelitis Optica Spectrum Disorders (NMOSD). When these genes were included in Gene Ontology analyses, pathways associated with CNS IDD syndromes were retrieved, suggesting that CHIKV-induced CNS outcomesmay share a genetic background with other neurological disorders. To our knowledge, this study was the first genome-wide investigation of genetic risk factors for CNS phenotypes in CHIKV infection. Our data suggest that HLA-DRB1 alleles associated with demyelinating diseases may also confer risk of CNS IDD outcomes in patients with CHIKV infection.

## Introduction

Chikungunya fever is typically described as a mild disease in which clinical symptoms are mainly related to articular pain and general malaise (Burt et al., 2017). Chikungunya virus (CHIKV) is an arthropod-borne virus from the genus *Alphavirus*, family *Togaviridae*, transmitted by the bite of *Aedes* mosquitoes and endemic in tropical countries (Pialoux et al., 2007). CHIKV infection is considered endemic in the Southeast Region of Brazil, with 1,159.4 cases/100,000 inhabitants having been registered in 2019 (BRASIL, 2020). However, involvement of central and peripheral nervous systems has been reported in 0.3 to 1% (Cerny et al., 2017; Anand et al., 2019), which are considered atypical manifestations of CHIKV.

The central nervous system (CNS) manifestations during CHIKV infections seem not to be different from other viruses like the Zika virus (Alves-Leon et al., 2019). White and gray matter are frequently affected in CNS cases and clinical findings sometimes mimic inflammatory demyelinating diseases (IDD) of the brain and spinal cord, mainly with clinical phenotypes of acute disseminated encephalomyelitis (ADEM), multiple sclerosis (MS), longitudinal extensive myelitis, multifocal myelitis, optic neuritis, and neuromyelitis optic spectrum disorders (NMOSD) (Mehta et al., 2018).

The immune process linked to CNS demyelination is triggered at least in part by post-infectious inflammation. The exact mechanism that leads to the development of these neurological manifestations in a small number of individuals with CHIKV infection is still unknown. In this context, ADEM and Myelitis may show similarities at clinical onset, such as disturbance of immune tolerance to myelin proteins and association with genetic variations mainly in HLA alleles, with presentation of MS, NMOSD and other non-MS autoimmune diseases of the CNS (Alves-Leon et al., 2009; Mowry and Waubant, 2010; Tobin et al., 2014; Lin et al., 2016; Höftberger and Lassmann, 2017).

Several viral and bacterial pathogens and different vaccines have been associated with ADEM and Myelitis (Menge et al., 2005; Yeshokumar and Waubant, 2017). Nevertheless, the discussion on whether CNS CHIKV manifestations are due to direct viral action or an autoimmune response triggered by the infection in a genetically susceptibility individual remains unclear. Variants in *TLR7* (rs179010) have already been associated with susceptibility to infection, while *TLR3* (rs6552950) was associated with disease severity and specific neutralizing antibody response (Her et al., 2015; Dutta and Tripathi, 2017). Other host genetic variants in *CD20913* and HLA alleles were associated with disease susceptibility or different clinical outcomes in CHIKV infection (Chaaithanya et al., 2016). Recently, patients with post-infectious neurologic syndromes (PINS) presenting mainly myelitis (frequency of 44%) and encephalomyelitis (frequency of 44%) were genotyped using the reference panel of the 1000 Genomes Project, but no risk alleles for MS were found (Martinelli-Boneschi et al., 2020).

The neurological manifestations of CHIKV infection make the differential diagnosis of immune-mediated PINS that affect the brain and spinal cord a challenge for neurologists. Since such outcomes are rare, it is not surprising that little is known about their genetic background. In regions such as Rio de Janeiro State, where CHIKV is highly incident, it is not unlikely that some CNS IDDs otherwise considered idiopathic, may, in fact, result from a previous or current CHIKV infection. Therefore, in order to better characterize the CHIKV-related CNS IDD phenotype, we herein provide a detailed clinical and radiological description of 6 patients presenting ADEM or Myelitis following CHIKV infection. Moreover, we performed whole exome sequencing (WES) analyses to investigate the genetic background and biological pathways associated with neurologic phenotypes of CHIKV infection.

## Materials and Methods

### Study Design and Patients

This is a prospective study, which included a convenience sampling of patients from an ongoing active cohort study in different hospitals in Rio de Janeiro and patients referred by Laboratório Central Noel Nutels (LACEN) in Rio de Janeiro, between July 2016 and June 2019. During this period, the health department from the Rio de Janeiro municipality registered 28,674 Dengue, 3,897 Zika and 51,626 Chikungunya cases with an increasing number of Chikungunya infections overcoming other arboviruses during the last 2 years 2018–2019. The cohort included 345 patients with 160 confirmed positive CHIKV infections investigated by direct detection of virus (RT-qPCR) or antibody detection (ELISA and PRNT evaluation). All participants were evaluated by a team of neurologists. When any neurological manifestation was detected, blood or cerebrospinal fluid (CSF) and imaging tests were requested to perform the differential diagnosis. Patient history, complete physical and neurological examination was performed, including details of serum and CSF testing, panel for autoimmune and other infectious diseases. Neuroimaging, nerve conduction studies, and electromyography were also used for classification of CNS or peripheral nervous system outcomes. After reviewing each case, patients who met the diagnostic criteria by use of standardized case definitions were classified as having CNS IDD phenotype. The other patients were classified without neurological manifestations or with other neurological diseases. Briefly, the patients diagnosed as ADEM fulfill diagnostic criteria (Sejvar et al., 2007; Krupp et al., 2013) of this type of IDD due the presentation of acute encephalopathy manifestations characterized by headache, epileptic seizures and mental confusion associated with brain and/or spinal cord white and gray matter lesions, some of them tumefactive. The diagnosis of Myelitis was based on development of sensory, motor, or autonomic dysfunction attributable to the spinal cord in conjunction with evidence of inflammation and exclusion of extra-axial compressive cause by neuroimaging in accordance with Transverse Myelitis Consortium Working Group (Transverse Myelitis Consortium Working Group, 2002). From our cohort the 6 patients (0.37%) who presented CNS IDD phenotype manifestations following CHIKV infections were selected for exome analysis. Six patients meeting the inclusion criteria who had no IDD were selected for exome analysis, as a baseline for comparisons. Individuals selected for this control group were paired by age and sex with the cases group. The study was approved by the local Ethics Committee (protocol number: 69411317.6.0000.5258) and a written informed consent was signed by all participants by the time of inclusion in the study. The demographic and systemic characteristics of CHIKV infection in these patients are described in Table 1. The CNS IDD manifestations included three cases of ADEM and three cases of Myelitis.

**Table 1:** Frequency of clinical, neurological manifestations, and in-hospital outcome.

	**CHIKV and CNS** ** IDD (*n* = 6) *N* (%)**	**CHIKV without CNS IDD** ** (*n* = 6) *N* (%)**
**Gender**		
Female	3 (50)	3 (50)
**Clinical findings**		
Fever	5 (83.3)	6 (100)
Rash	2 (33.3)	5 (83.3)
Myalgia	4 (66.6)	6 (100)
Arthralgia/itis	3 (50)	6 (100)
Headache	5 (83.3)	1 (16.6)
Retroorbital pain	0	3 (50)
**Outcome**		
Death	0	0

### Neuroimaging Diagnosis

Magnetic resonance imaging (MRI) was performed using 1.5 or 3.0 Tesla scanners, depending on the clinical availability. The protocols included brain, cervical and spinal cord MRI according to clinical symptoms. Brain MRI included 3-dimension fluid-attenuated inversion-recovery sequences (3D FLAIR), 3D T1 weighted-images (WI), T2-WI, diffusion WI (DWI), short-tau inversion recovery (STIR) for optic nerve analysis. Spinal cord MRI included sagittal STIR, T2-WI, and T1-WI and axial T2-WI. Brain and spinal cord fat suppressed (FS) T1-WI post-gadolinium scans were also obtained, on axial and sagittal planes.

### Chikungunya Diagnosis

Blood and/or urine samples were collected at patient admission. Cerebrospinal fluid was also collected from patients with CNS IDD and stored at −80°C until further analysis. Biological samples were blindly tested for Zika (ZIKV), CHIKV, and Dengue (DENV) virus. Samples selected for exome analysis had their molecular and serological diagnosis confirmed as described below.

Virus RNA was obtained from serum, urine or CSF samples using the QIAmp MinElute Virus Spin Kit (Qiagen), according to manufacturer's instructions. RNA was eluted in 50 and 5 μL were used for virus genome detection using the Go-Taq Probe 1-step RT-qPCR System (Promega) and previously designed primers targeting CHIKV, DENV, and ZIKV (Lanciotti et al., 2007, 2008; Menting et al., 2011). Antibodies against CHIKV infection (IgG and IgM) were investigated by MAC-ELISA (Euroimmun) using serum samples, according to manufacturer's instructions. Plaque-reduction neutralization tests (PRNT) were also performed to detect neutralizing antibodies against CHIKV and to exclude cross-reaction with other arboviruses circulating in the same region. Briefly, plasma samples were incubated at 58°C for 30 min to inactivate CHIKV and limit complement effect. Serial dilutions (from 1:5 to 1:2,560) of heat-inactivated plasma were incubated with an equal volume containing 100 plaque forming units (PFU) of CHIKV isolate BHI3745/H804709 (Accession Number KP164570.1) for 1 h at 37° C. The virus-sera mixture was inoculated into confluent monolayers of VERO cells. After 1 h, inoculum was removed and semisolid medium (1.2% carboxymethylcellulose in alpha-MEM supplemented with 1% fetal bovine serum) was added. Cells were further incubated for 30 h and then fixed with 4% formaldehyde solution. Cells were stained with crystal violet dye solution. PRNT end-point titers are expressed as the reciprocal of the last serum dilution showing reduction in plaque counts ≥90%. PRNT_90_ titer ≥20 was considered positive for the presence of neutralizing antibodies against CHIKV.

### DNA Extraction and Whole Exome Sequencing

Genomic DNA (gDNA) was obtained from blood cells using the MasterPure Complete DNA and RNA Purification Kit (Lucigen). Integrity of gDNA was accessed by agarose gel electrophoresis and quantification was performed using the Qubit dsDNA HS Assay (Thermo Fisher Scientific). Libraries were prepared using Illumina TruSeq Exome Library Prep kit according to the manufacturer's protocol. Sequencing was performed with the Illumina NextSeq® 500/550 High Output Kit v2 (150 cycles), generating 2 × 75 bp paired-end reads. Raw sequencing data files in FASTQ format generated in the Illumina platform were preprocessed in fastqc and trimmomatic for quality assessment and trimming analysis, respectively. Sequenced reads were aligned to the GRCh38 human reference genome using Burrows-Wheeler Aligner (BWA) (Li and Durbin, 2009). Additional BAM file manipulations were performed with Samtools (Li et al., 2009) for sorting and mapping quality filtration (Q30). Duplicate reads were marked using Picard MarkDuplicates tool (http://broadinstitute.github.io/picard). Base quality recalibration and variant calling were performed using BQSR and HaplotypeCaller from Genome Analysis Toolkit (GATK) (McKenna et al., 2010).

### Detection of Pathogenic and Potentially Pathogenic Variants

In order to select potential pathogenic variants, we establish two sets of genes. First, a panel of genes previously related to CHIKV infection and neurological impairment was established based on a literature review. We next extended the gene panel by retrieving genes related to enriched Gene Ontology (GO) terms such as pathogen response, immune response, metabolic regulation and expression regulation. To investigate the relationship between the two sets of genes, a connectome analysis was performed (Itan et al., 2013) by estimating the pairwise protein-protein interaction between genes. A *p* ≤ 0.05 was applied to filter interactions. Next, the Mendelian inheritance model was retrieved for each gene by querying the OMIM database. Finally, we prioritize possibly damaging variants mapped in the final gene list for each patient (Supplementary Figure 1).

Gene-based variant findings were further filtered following functional annotation. Only protein-altering variants were selected, including truncating variants (stop gain/loss, start loss, or frameshift), missense variants, canonical splice-site variants, and inframe indels affecting protein-coding regions. Overall, these functional annotations correspond to high and moderate impact annotation. Variant pathogenicity was predicted using computational tools such as SIFT (Sorting Intolerant from Tolerant) (Ng and Henikoff, 2003), PolyPhen (Polymorphism Phenotyping) (Adzhubei et al., 2013), CADD (Combined Annotation-Dependent Depletion) (Kircher et al., 2014), and LoFtool (Fadista et al., 2017) according to the Ensembl Variant Effect Predictor database (VEP) (McLaren et al., 2016). In addition, American College of Medical Genetics and Genomics (ACMG) (Richards et al., 2015) and ClinVar (Landrum et al., 2018) recommendation guidelines were also assigned. After these filtration steps, we researched the population frequency of the variants in 1000 Genomes Project, Exome Aggregation Consortium (ExAC) and Genome Aggregation Database (gnomAD). The analysis pipeline is shown in Supplementary Figure 2.

### HLA Typing From Exome Data

The HLA alleles were predicted from WES reads with the HLAminer tool (Warren et al., 2012). Sequences in FASTQ format were mapped against chromosome 6 (GRCh38) using bowtie2 (Langmead and Salzberg, 2012). To predict HLA alleles, genomic sequences of classes I and II HLA were retrieved from the IMGT/HLA database in FASTA format. WES sequences were assembled into 200 bp contigs using the TASR tool (Warren and Holt, 2011). Contigs were aligned to HLA reference sequences using BLAST (Basic Local Alignment Tool). Aligned sequences were then used to predict HLA alleles.

### Pathway Enrichment Analysis

The biological significance of the filtered gene set was explored by enrichment analysis of the GO term for the biological process. Enrichment analysis was conducted using the ClusterProfiler R package v.3.18.0 (Yu et al., 2012) to produce gene clusters (≥2 genes/cluster). We filtered GO terms with a *p*-value and *q*-value (Benjamini & Hochberg (BH) method) smaller than 0.05.

## Results

This is a series case report describing clinical and neurological outcomes and exome analysis associated with CHIKV infection. In the following sections we describe the neuroimage findings in the CHIKV-confirmed patients followed by the genetic variants and cellular pathways in patients harboring CNS outcomes.

### Central Nervous System IDD Phenotypes in Patients With CHIKV Infection

**Figure 1:**
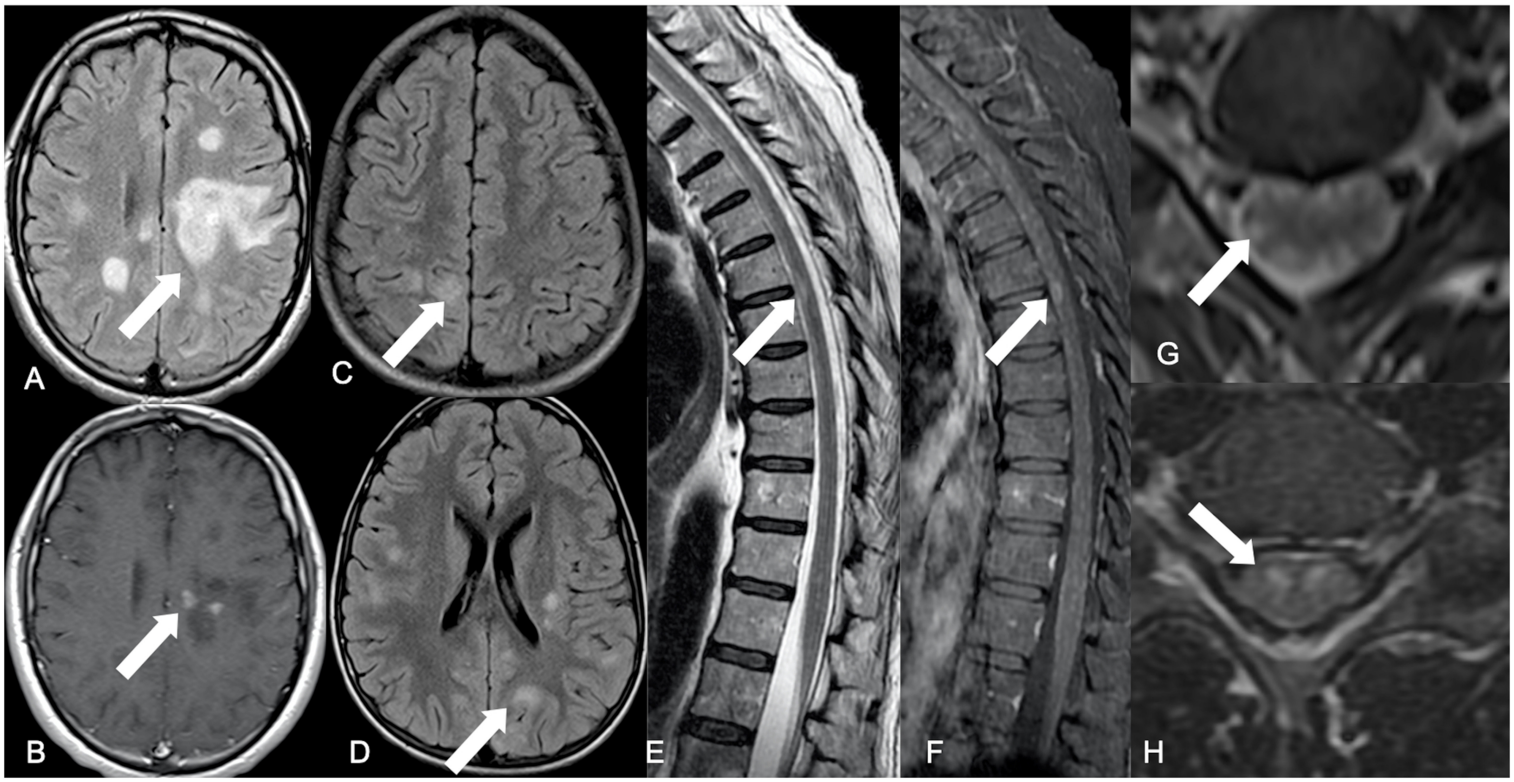
Summary of main neuroimaging findings. Brain axial FLAIR showing a tumefactive hyperintense lesion located in the frontal-parietal transition of the left hemisphere (white arrow) **(A)**, with punctate discontinued contrast enhancement on the corresponding axial post-contrast T1-WI (white arrow) **(B)**, representing an ADEM pattern. An encephalitis pattern can be seen on brain FLAIR axial images (white arrows) with cortical-subcortical hyperintense diffuse lesions **(C,D)**. Dorsal spinal cord sagittal T2-WI with elongated areas of peripherally located hypersignal intensity among the white matter tracts (white arrow) **(E)**, associated with contrast enhancement on sagittal FS T1-WI (white arrow) **(F)**. Axial T2-WI have elongated hypersignal images in a radial distribution, from the central spinal cord gray matter to the peripheral white matter (white arrow) **(G)**. Axial T2-WI with hypersignal affecting the gray matter anterior horn, in a polio-like pattern (white arrow) **(H)**.

During the period of this study, our multidisciplinary team assisted 345 patients, 160 of which had CHIKV infection, and the remaining were diagnosed with either Dengue or Zika infection. Within the CHIKV positive patients, sixteen presented involvement of the nervous system, and six were classified as having neurological manifestations mimetizing CNS IDD. For all patients that we perform the exome (with and without IDD), the most frequent systemic arbovirus symptoms overall were fever [11 (92%)], myalgia [10 (83%)], and arthralgia [9 (75%)]. Interestingly only one patient with IDD phenotype following CHIKV infection was completely asymptomatic for the classic signs and symptoms of the infection. These patients never had any previous demyelinating manifestations. All patients with CNS IDD phenotype underwent imaging which showed the extent of the inflammatory lesion (Figure 1, Supplementary Figures 3–8). Clinical and radiological characteristics are summarized in Table 2 and detailed in Supplementary Table 1, which shows all clinical, infectious and neurological features of each patient, as well as the exams at the time of the neurological condition.

**Table 2:** Neurological manifestations associated with CNS IDD phenotype diagnosis.

**Patient**	**Neurological features**	**Neurological diagnosis**
Case 1 (18ZC026)	Progressive numbness and paresthesia involving LLE up to T8 level and RLE up to S1 level. LE hyperreflexia. Preserved power. Superficial abdominal and plantar reflexes absent on the L, normal response on the R. GCS 1;5 Normal CSF; Intramedullary signal abnormality with GAD enhancement at T4-5 level	Myelitis
Case 2 (19ZC131)	Headache acutely worse; normal head CT + CSF with mononuclear pleocytosis and low glucose. Developed urinary incontinence; billateral lower extremity weakness and blurred vision. Repeat CSF showed increased MN cellularity and normal glucose. Fundoscopy showed papilledema and brain MRI showed asymmetrical signal abnormalities involving bl. Cortico–subcortical regions, white matter, and corpus callosum	ADEM
Case 3 (19ZC147)	Quadriparesis, worse in LE and high frequency tremor affecting whole body, worse in upper extremities. CSF showed only increased protein. Developed LE hyperreflexia and bilateral plantar reflex extension, without objective sensory level. MRI spine showed signal abnormalities with contrast enhancement	Myelitis
Case 4 (19ZC160)	Paraplegia evolving with dysarthria, urinary incontinence and left arm paresis. Hyperreflexia of the right patellar reflexes, deviation oh the left labial commissure	ADEM
Case 5 (19ZC161)	Overall reduction in muscle tone, urinary retention, evolving in 20 days with paraplegia	Myelitis
Case 6 (19ZC179)	Persistent fever and headache presented acutely with myoclonic jerks, altered mental status, mutism, rigidity, trismus, and possible tonic seizure. Evolved with aspiration pneumonia. CPK was increased at presentation, possibly secondary to immobility or seizure. Urinary retention and quadriparesis with hyperreflexia, brisk deep abdominal reflexes with absent superficial abdominal reflexes, multidirectional nystagmus, and diplopia followed. Head CT was normal, head MRI showed meningeal contrast enhancement with signal abnormalities at the rhombencephalon and cervical MRI showed signal abnormalities. CSF showed increased protein and monocytic pleocytosis. Interictal EEG showed only diffuse slowing with sedation and AED	ADEM

### Identification of Genetic Variants, Functional Annotation of Genes in Exomes Associated With CNS IDD

**Figure 2:**
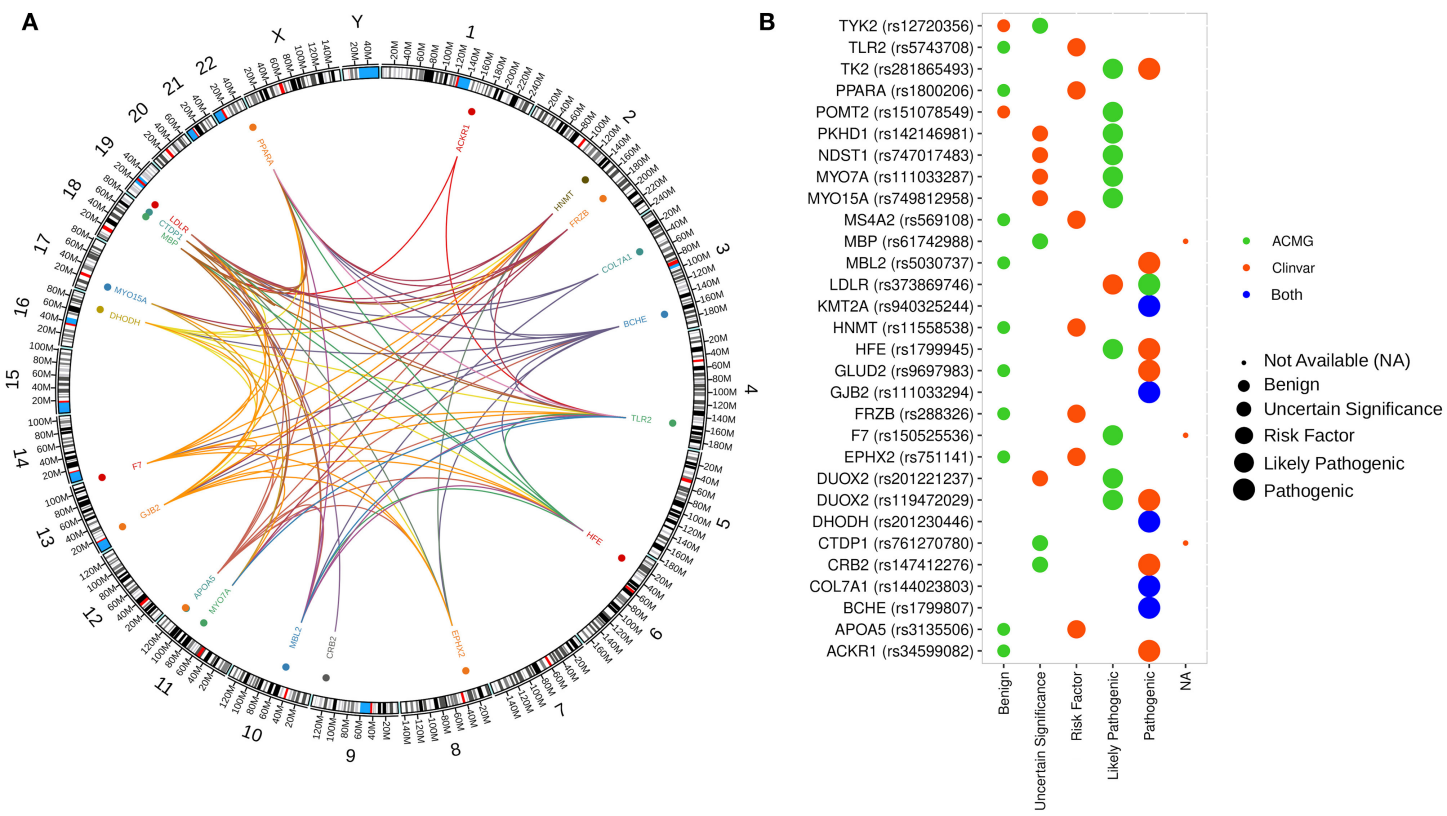
Distribution and interaction between genetic variants and gene ontology across the human genome. **(A)** Genomic map showing the distribution of genes with predicted pathogenic variants selected by our analysis. Colored dots represented the localization of genes with at least one variant in all patients. The innermost layer represents the connections between the functional annotation of the genes. **(B)** Pathogenicity classification of genetic variants from our study according to ClinVar and ACMG guideline recommendations.

On average, 98% of the reads from the WES mapped to the reference genome. Targeted exonic regions achieved 121-fold mean sequence coverage with 91% of bases covered at >20-fold. Genetic variants were filtered based on the impact in protein changes, computational predictions of pathogenicity and population frequency. Overall, we interrogated variants with deleterious profiles according to different algorithms (SIFT, PolyPhen, CADD, and LoFtool), low frequency variants in databases from the 1,000 Genomes Project, ExAC, or gnomAD and variants with pathogenic profiles according to ACMG and ClinVar recommendations. In total, a set of 30 promising variants was defined (Figure 2, Supplementary Table 2). Specifically, in the 6 neurologically affected patients, we found 24 variants in 23 genes, 20 were found exclusively in these patients, while four variants were also found among controls. Most of the identified variants have minor allele frequencies lower than 0.01, and we were unable to identify any variant that explains the CNS IDD manifestations when compared with the unaffected group or a cohort of 609 aged Brazilian individuals (Naslavsky et al., 2017).

A phenotype-based analysis allowed the identification of 4 genes (*TLR2, MBP, TYK2*, and *CTDP1*) related to CHIKV infection with neurological involvement that harbor variants with pathogenicity prediction. TLR2 is a pattern-recognition receptor associated with cell-mediated immunity, which has been associated mainly with recurrent mycobacterial infection. A role in neurogenesis has also been described (OMIM #607948). The *MBP* gene encodes a transmembrane IgE receptor associated with susceptibility to multiple sclerosis (OMIM#126200) and the *TYK2* gene encodes a tyrosine kinase associated to type I and III interferon signaling, playing a role in antiviral immunity. Deficiency in TYK2 is associated with Immunodeficiency 35 phenotype and increasing susceptibility to viral infection (OMIM#611521). *CTDP1* has been associated with congenital cataract, facial dysmorphism, and neuropathy (OMIM#604168) phenotypes. Notably, variant rs5030737 [c.154C>T, p.Arg52Cys] in *MBL2*, previously associated with mannose-binding lectin deficiency and chronic infections (OMIM#614372), was also detected in a CNS IDD case.

Afterwards, classic HLA alleles [HLA-A, HLA-B, HLA-C, HLA-DP (DPA1, DPB1 and DPB2), HLA-DQ (DQA1, DQA2 and DQB1) and HLA-DR (DRA, DRB1, 3, 4, and 5)] were predicted from WES data for each individual, along with non-classic HLA-G, HLA-E, and HLA-F alleles (Supplementary Table 3, Supplementary Figure 2).

To briefly describe the main variations found in each patient with CNS IDD phenotypes, we highlight that case 1 (18ZC026) harbored four pathogenic, missense variants at *PKHD1, TYK2, FRZB*, and *HNMT* genes, in addition to the haplotype DRB3^*^02:02-DQA1^*^05:01, associated with susceptibility to Graves' disease (Chen et al., 2000), and DQA1^*^05:01-DQB1^*^02:01-DRB1^*^03:01 haplotype, associated with risk of NMOSD (Zéphir et al., 2009; Brum et al., 2010; Deschamps et al., 2011) (Table 3, Supplementary Table 2). Case 2 (19ZC131) has eight missense variants within several genes, including Gly407Ser at *LDLR*, associated with familial hypercholesterolemia 1 phenotype (OMIM#143890) and also the rs5030737 polymorphism in the *MBL2* gene, previously associated with mannose-binding lectin deficiency and chronic infections (OMIM#614372). This patient also carries DRB1^*^15 and DPA1^*^03:01 HLA alleles, which were significantly associated with ADEM (Alves-Leon et al., 2009), and haplotype DRB3^*^02:02-DQA1^*^05:01, associated with Graves' disease (Chen et al., 2000; Table 3, Supplementary Table 2).

**Table 3:** Distribution of Class II HLA alleles by CHIKV-related CNS IDD cases.

**Case 1** ** (18ZC026)**	**Case 2** ** (19ZC131)**	**Case 3** ** (19ZC147)**	**Case 4** ** (19ZC160)**	**Case 5** ** (19ZC161)**	**Case 6** ** (19ZC179)**
DPA1*01:03/ DPA1*02:01	DPA1*01:03/ DPA1*03:01	DPA1*01:03	DPA1*01:03	DPA1*02:01	DPA1*01:03/ DPA1*02:01
DPB1*04:01/ DPB1*57:01	NA	DPB1*04:01	DPB1*04:01	NA	NA
DPB2*01:01	DPB2*01:01/ DPB2*03:01	DPB2*03:01	DPB2*03:01	DPB2*03:01	DPB2*01:01/ DPB2*03:01
DQA1*02:01/ DQA1*05:01	DQA1*05/ DQA1*03	DQA1*05:01	DQA1*01:02^^#^^	DQA1*02:01/DQA1*05:01	DQA1*01:07
DQA2*01	DQA2*01:05	DQA2*01	DQA2*01	DQA2*01	DQA2*01
DQB1*02:01	DQB1*03:01	DQB1*03:01/DQB1*02:01	DQB1*06:02^^#^^	DQB1*02:01/ DQB1*03:01	DQB1*05:02
DRA*01:01	DRA*01:01	DRA*01:01	DRA*01:01	DRA*01:01	DRA*01:01
DRB1*03:01/ DRB1*07:01^^#^^	DRB1*15/ DRB1*12^^#^^	DRB1*03:01/ DRB1*11:01^^#^^	DRB1*15:01^^#^^	DRB1*11:02/ DRB1*07:01^^#^^	DRB1*16:02/ DRB1*01:01^^#^^
DRB3*02:02	DRB3*02:02	DRB3*02:02	NA	DRB3*02:02	NA
DRB4*01:01	DRB4*01:01	NA	NA	DRB4*01:01	NA
NA	NA	NA	DRB5*01:01^^#^^	NA	DRB5*02:02/ DRB5*01:01^^#^^
**Main haplotypes identified**					
DRB3*02:02-DQA1*05:01^a^	DRB3*02:02-DQA1*05:01^a^	DRB1*11:01-DQA1*05:01-DQB1*03:01^c^	DRB1*15:01-DQA1*01:02-DQB1*06:02-DRB5*01:01^d^	DQA1*05:01-DQB1*02:01^e^	DRB5*01:01-DRB1*16:02^f^
DQA1*05:01-DQB1*02:01-DRB1*03:01^b^				DRB3*02:02-DQA1*05:01^a^	

*NA, Not Available*.

# *Alleles related to neurological manifestations such as inflammatory demyelinating disease*.

a *Associated with Graves' disease (Chen et al., 2000)*.

b *Associated with risk of NMOSD (Zéphir et al., 2009; Brum et al., 2010; Deschamps et al., 2011)*.

c *Associated with Lupus erythematosus (Miyagawa et al., 1997*).

d *Associated with central nervous system demyelination and autoimmune disease (Dyment et al., 2005; Kaushansky and Ben-Nun, 2014; Mack et al., 2019)*.

e *Associated with celiac disease (Selleski et al., 2018; Farina et al., 2019)*.

f *Associated with systemic lupus erythematosus (Louthrenoo et al., 2013)*.

The missense variant (Arg376Trp), located in the *DUOX2* gene, was found in cases 2 and 3 (19ZC147). The *DUOX2* gene is associated with thyroid dyshormonogenesis 6 phenotype (OMIM#607200). Case 3 also carries a variant in the *CRB2* gene, the HLA-DRB1^*^03 allele related to NMOSD (Zéphir et al., 2009; Brum et al., 2010; Deschamps et al., 2011), and haplotype DRB1^*^11:01-DQA1^*^05:01-DQB1^*^03:01, associated with lupus erythematosus (Miyagawa et al., 1997; Table 3, Supplementary Table 2). Case 4 (19ZC160) has five missense variants, including His63Asp, within the *HFE* gene, involved in iron absorption and associated with hemochromatosis (OMIM#235200) and microvascular complications of diabetes 7 (OMIM#612635). Variants in the *TK2* gene, associated with mitochondrial DNA depletion syndrome 2 (OMIM#609560), and in the *MBP* gene, associated with susceptibility to multiple sclerosis (OMIM#126200), were also found. Case 4 was also homozygous for variant rs9697983 [c.1492T>G, p.Ser498Ala] in the *GLUD2* gene, associated with Parkinson's disease (OMIM#168600). Patient 19ZC160 also carries the HLA haplotype DRB1^*^15:01-DQA1^*^01:02-DQB1^*^06:02-DRB5^*^01:01, associated with CNS demyelination and autoimmune disease (Dyment et al., 2005; Kaushansky and Ben-Nun, 2014; Mack et al., 2019), and the DPA1^*^01:03 and DPB1^*^04:01 alleles, associated with narcolepsy (Cortes et al., 2004; Louthrenoo et al., 2013; Table 3, Supplementary Table 2).

Case 5 (19ZC161) has four missense variants, including rs751141, in the hydrolase *EPHX2* gene, associated with hypercholesterolemia (OMIM#143890), and rs761270780, in the *CTDP1* gene. Moreover, HLA haplotype DQA1^*^05:01-DQB1^*^02:01 and DRB3^*^02:02-DQA1^*^05:01, associated with celiac disease (Selleski et al., 2018; Farina et al., 2019) and Graves' disease (Chen et al., 2000), were also found (Table 3, Supplementary Table 2). Case 6 (19ZC179) had two variants, including rs5743708, in *TLR2*. This patient also carries the HLA-A^*^03 allele, associated to MS (Naito et al., 1972), B^*^51:01/B^*^27:05 alleles, associated with inflammatory disease (Mizuki et al., 1997), DQB1^*^05:02, associated with Myasthenia gravis (Testi et al., 2012), and the DRB5^*^01:01-DRB1^*^16:02, associated with susceptibility to systemic lupus erythematosus (Louthrenoo et al., 2013; Table 3, Supplementary Table 2).

Despite the lack of significantly overrepresented pathways in patients with CHIKV-associated neurologic outcomes, we found that the associated set contains 29 genes annotated by at least one of the 24 GO terms enriched at *q* < 0.05 (Figure 2, Supplementary Table 4). The enriched GO includes pathways already related with IDD syndrome, such as leukocyte activation involved in the inflammatory response (GO:0002269; *p* = 2.51E-3) and microglial cell activation (GO:0001774; *p* = 2.51E-3).

## Discussion

In the last decades, the idea of a virus playing a role in the development of IDD has been reinforced in several studies (Lucas et al., 2011). It is believed that infections can trigger such demyelinating lesions by the activation of autoreactive T cells, with primary events taking place outside the CNS further initiating pathogenic T cell reactivation within the CNS, or by molecular mimicry and developing an autoantibody response (Salvetti et al., 2009; Delogu et al., 2011; Anaya et al., 2016; Burnard et al., 2017). Here, we describe ourselves for the first time that CHIKV patients who developed CNS IDD phenotype shared a common genetic signature with diseases such as MS and NMOSD.

During CHIKV infection progression viremia is observed up to the 5–7 days post-illness onset (pio), whereas IgM can be detected from 2 days pio up to 4 months, and IgG from 4 days pio to over one year (Tanabe et al., 2018). By the time of neurological evaluation, all patients described herein were both CHIKV IgM and IgG reactive, whereas presenting a prodrome period varying from 7 to 30 days (from CHIKV-like symptoms to neurological ones), suggesting they were either at the acute phase of the infection, or at the early-chronic phase of the infection. Two important characteristics of nervous system involvement in CHIKV infection are the fact that it is rare and that it can mimic autoimmune inflammatory diseases in adults. Among CHIKV positive patients, sixteen presented nervous system involvement and six were classified as having neurological manifestations mimetizing CNS IDD as ADEM and extensive transverse myelitis. Despite the relatively small sample size, literature shows that 0.3–1% of cases will develop severe neurological manifestations, corroborating our findings (Cerny et al., 2017).

The genome-wide search for genetic variants associated to diseases such as MS, NMOSD, ADEM, and myelitis has suggested that the sharing of molecular pathways between these immune-mediated diseases may be greater than we imagined (Oldstone, 1998; Kakalacheva et al., 2011; Kattimani and Veerappa, 2018; Zamvil et al., 2018). This justifies similar heterogeneous clinical phenotypes associated with various environmental factors, such as the seasonal arbovirus infections that Brazil and other countries have been exposed to. In this context, the viral trigger is an opportunity to investigate the encounter between individual genetic variants that may be associated with the immune response and CNS IDD. Possible explanations to these questions include the phenomenon of molecular mimicry and shared polymorphisms between these immune mediated diseases, both hypotheses to be further explored.

Although we have not identified any variant that could explain susceptibility to IDD syndromes following CHIKV infection in our cohort, these results do not exclude a genetic component for this neurological manifestation. Indeed, the lack of a clear genetic cause reinforces the hypothesis of a multifactorial inheritance. The most significant GO terms were related to regulation of chemokine production (GO:0032642; *p* = 2.75E-4), endocytosis (GO:0030100; *p* = 8.87E-4), acute inflammatory response (GO:0006953; *p* = 2.41E-3) and leukocyte activation involved in inflammatory response (GO:0002269; *p* = 2.51E-3). They all reflect mechanisms of viral infection, endocytosis and immune mediated inflammatory response (Lindenbach and Rice, 2003; Gao et al., 2020). Interestingly, we also found evidence for microglial cell activation (GO:0001774; *p* = 2.51E-3) only in patients with ADEM after CHIKV, which is involved in immune regulation and CNS autoimmune disease (Dheen et al., 2007; O'Loughlin et al., 2018). Neuroinflammation is a complex integration of the responses of all cells present within the CNS and consequent microglial activation and leukocyte infiltration (Carson et al., 2006).

The demyelinating lesions found in most MS and NMOSD patients are mainly associated with T cell inflammation, with the involvement of both microglia and infiltrating peripheral macrophages - as revealed by TMEM119 immunohistochemical profiling - in the pathology of the lesions (Popescu and Lucchinetti, 2012; Zrzavy et al., 2017; Hayashida et al., 2020). Interestingly, infection of the CNS by the CHIKV has been linked to microglial activation (Ganesan et al., 2008; Abere et al., 2012). The overproduction of inflammatory modulators and oxidative stress may induce mitochondrial dysfunction of oligodendrocytes in demyelinating diseases, such as MS, that may cause irreversible cell damage and consequently play a key role in CNS demyelination (Liñares et al., 2006; Venugopalan et al., 2014; Banerjee and Mukhopadhyay, 2018; Colavita et al., 2018; Lan et al., 2018). Recently, the International Multiple Sclerosis Genetics Consortium performed a large genetic association study, which identified an enrichment of genetic variants in human microglia but not in astrocytes or neurons (International Multiple Sclerosis Genetics Consortium, 2019).

Large GWAS studies have identified the tyrosine kinase 2 (*TYK2*) gene as associated with susceptibility to MS (Ban et al., 2009; Mero et al., 2010). In patient 18ZC026 (myelitis), we found the rs12720356 variant in the *TYK2* gene. Of note, this SNV introduces a missense mutation (I684S) in the catalytic domain, which maintains residual function in response to IFN-α/β and other pro-inflammatory cytokines. It is known that TYK2 kinase impaired activity impacts type I IFN signaling and alters defense against the virus (Prchal-Murphy et al., 2012). This non-synonymous variant might decrease IFN-I signaling, hindering anti-viral immunity, and if associated with HLA alleles may be related to the development of IDD.

Noteworthy, HLA class II alleles have been linked to a wide range of autoimmune diseases (Svejgaard, 2008; Asgari et al., 2012; Bodis et al., 2018). Here, we found the DR15 haplotype (DRB1^*^15:01-DQA1^*^01:02-DQB1^*^06:02) and the HLA-DRB5^*^01:01 allele (DR2) in patient 19ZC160, while at least one of the alleles related to these haplotypes was found in the other ADEM patients. It is important to highlight that we found the HLA-DRB1^*^15:01 allele only in patients with ADEM, which is clinically cataloged as a risk factor for MS (VCV000029757). The literature reports that ADEM can progress similar to MS and these HLA haplotypes are the main genetic risk factor that contribute to the etiology of MS (Schmidt et al., 2007; Etzensperger et al., 2008).

Some authors have already described that the presence of certain HLA alleles may be related to the development of ADEM, especially in childhood (Ramachandran Nair, 2004; Waubant et al., 2016). Patient 19ZC179 carries HLA-DRB1^*^01:01 and HLA-DRB5^*^01:01 alleles, which were associated with ADEM in Russian and Korean populations (Idrissova et al., 2003; Oh et al., 2004). We also identified the HLA-DRB1^*^15:03 allele in patient 19ZC129 without neurological manifestation. This allele has been associated with ADEM in a small case-control study in Brazil, but results still lack replication in larger cohorts (Alves-Leon et al., 2009).

Two of the 3 patients who developed myelitis associated to CHIKV infection carry the HLA-DRB1^*^03:01 allele, linked to NMOSD in Brazilian patients from Rio de Janeiro (Alvarenga et al., 2017), whereas that same allele was identified in only one individual without neurological manifestations. It is important to note that HLA-DRB1^*^03:01 has also been associated to susceptibility to rheumatoid arthritis, mainly when inherited together with HLA-DRB1^*^09:01, which is the case of this patient (van Drongelen and Holoshitz, 2017). In addition, the HLA-DRB1^*^09:01 allele has already been reported as a protective factor for NMOSD in a Japanese population (Matsushita et al., 2020).

All patients who developed myelitis following CHIKV infection carried the HLA-A^*^23:01 allele, whereas it was identified in only one individual without neurological manifestation. The distribution of HLA-A^*^23:01 has been analyzed in different Brazilian regions and throughout the world, specifically in different ethnic groups from Rio de Janeiro, and an allele frequency lower than <0.1 has been reported (http://allelefrequencies.net). On the other hand, alleles already reported in the literature as protectors against autoimmune diseases were found only in patients without IDD. For example, HLA-B^*^44:02 is a protective allele for MS susceptibility, associated with better MRI outcomes in terms of brain parenchymal fraction and T2 hyperintense lesion volume (Healy et al., 2010). Of particular interest, HLA-DRB1^*^13 alleles are the protective alleles shared by multiple autoimmune diseases and it has already been observed that HLA-DRB1^*^13:02 confers protection against organ-specific autoimmune diseases and some infectious diseases (Furukawa et al., 2017).

The variant that most caught our attention was rs61742988 (*MBP*) in patient 19ZC160, who also carries HLA DR15 and DR2 haplotype. To our knowledge, the rs61742988 variant has been described in few publications and classified as having uncertain significance, according to the ACMG guideline (Richards et al., 2015). Independent studies have also shown this mutation in the *MBP* gene to be associated to clinical course in MS (Zhou et al., 2017) and, in association with HLA-DRB1^*^15:01, the allele may be considered a predisposing factor for this disorder (Nejati et al., 2017).

Candidate gene approaches or GWAS studies have not been able to clearly establish any markers that predict susceptibility or clinical course after CHIKV infection. Indeed, different disease outcomes are like multifactorial traits, in which phenotypes is determined by the interaction between several genes, environment factors and epigenetic mechanisms (Hill, 2006; Oksenberg and Baranzini, 2010; Paschos and Allday, 2010; Ho et al., 2012; Ladd-Acosta and Fallin, 2016). The exact mechanisms whereby CHIKV infection influences CNS IDDs are not yet well-established and we suggest that in genetically susceptible individuals, molecular mimicry could induce myelin-reactive T cells or autoantibody production, resulting in cross-reaction to both viral and myelin proteins (Oldstone, 1998).

We know there are more than 200 genetic variants in coding and non-coding regions of DNA that are related to susceptibility or progression of MS (Cotsapas and Mitrovic, 2018) and MS genetic burden (MSGB) score, one of the most used concepts today, is based on a weighted scoring algorithm using SNPs associated with MS (Isobe et al., 2016). Therefore, one of the important limitations of our study is related to the non-identification of genetic variants in non-coding regions.

Regarding genotype-phenotype correlations, the most interesting finding in the present study was the specific HLA-DRB1 allele conferring a similar phenotype to MS and NMOSD. Taken together, our data suggest a role for HLA alleles and candidate genes in the development of demyelinating events following CHIKV infection. Further analyses in larger cohorts are required to replicate and functionally validate these results. Questions that still need to be answered include whether exposure to new viral antigens in populations around the world, through deforestation, species migrations and environmental changes, may impact the incidence of autoimmune diseases in the future.

## Data Availability Statement

The WES dataset used in our study is publicly available in SRA-NCBI (www.ncbi.nlm.nih.gov/sra), SRA accession PRJNA680041.

## Ethics Statement

The studies involving human participants were reviewed and approved by Research Ethics Committee of HUGG/UNIRIO (protocol number: 69411317.6.0000.5258) and a written informed consent was signed by all participants by the time of inclusion in the study. Written informed consent to participate in this study was provided by the participants' legal guardian/next of kin.

## Author Contributions

SVA-L: designed the research clinical and data information, neurological evaluation, data analysis, and drafted the manuscript. CdSF and RdSFJ: exome data analysis and drafted the manuscript. ALH: blood samples analysis and initial draft of the manuscript. FLF-D: recruited patients and sorted out clinical information, collected the blood samples, data analysis, and drafted the manuscript. FCR-L: acquisition, analysis of radiological image data, and drafted the manuscript. JPdCG and ADdA: recruited patients and sorted out clinical information, neurological evaluation, and collected the blood samples. CCdSR: recruited patients and sorted out clinical information and neurological evaluation. LMH: blood samples analysis. ALG and APdCG: exome sequencing. MTdM, MCdPT, and RAM: blood samples analysis. BMGN and LCF: collected the blood samples. MMdS, CN, ASdSC, and CCFV: recruited patients and sorted out clinical information, and neurological evaluation. AT: review of the manuscript. OCFJ and RSA: blood samples analyses and review of the manuscript. CCC: data analysis and drafted the manuscript. ATRdV: coordinated the exome experiments, data analysis, drafted the manuscript, and critical review of the manuscript. All authors contributed to the article and approved the submitted version.

## Conflict of Interest

The authors declare that the research was conducted in the absence of any commercial or financial relationships that could be construed as a potential conflict of interest.

## Supplementary Material

Supplementary Figure 1 Flowchart of gene panel prioritization. Strategies of variant filtration based on gene panel selection for CHIKV infection and neurological impairment. The main genes related to CHIKV infection, Myelitis and ADEM were firstly retrieved from the literature. Next, we expanded the gene set by retrieving genes related to enriched GO terms. To further investigate the relationship between the two sets (genes from the literature and recovered from GO) a pairwise protein-protein interaction (connectome) was performed. We retrieved the mendelian inheritance model for each gene by querying the OMIM database. Finally, we prioritize possibly damaging variants mapped in the final genes list for each patient.

Supplementary Figure 2 Flowchart of the pipeline used to filter genetic variants and predict HLA alleles. From fastq files originated by WES, the pipeline was subdivided into two main approaches: (i) variant prioritization and (ii) HLA allele prediction. The set of variants selected was analyzed sequentially using computational tools to predict pathogenicity. They were then filtered by low frequency using population databases and further classified according to clinical guidelines from pathogenicity databases. Prediction of HLA alleles (class I and II) was performed with the HLAminer tool.

Supplementary Figure 3 Central nervous system MRI of case 1. Sagittal STIR **(A)** and T1 FS post-contrast **(B)** and sagittal T2 **(C)** and T1 FS post-contrast **(D)** of the cervical and dorsal spinal cord respectively show sparse hyperintense focci (white arrows), some with gadolinium enhancement, notably in the cervical-dorsal transition, extending <1 vertebral body, mostly affecting the posterior columns on axial T2 WI (white arrows) **(E,F)**, resembling MS like pattern dissemination.

Supplementary Figure 4 Central nervous system MRI of case 2. Brain axial FLAIR images **(A–C)** show hyperintense cortical-justacortical lesions identified in the parietal and occipital lobes (white arrows), in the periventricular white matter and adjacent to the occipital horns of the lateral ventricles, characterizing an encephalitis pattern.

Supplementary Figure 5 Central nervous system MRI of case 3. Dorsal spinal cord sagittal T2 WI **(A)** have multiple peripheral hypersignal intensity focci (white arrows), with contrast enhancement on FS post-contrast T1 WI **(B)**. Tiny focci of hypersignal intensity peripherally located on cervical axial T2 WI **(C,D)**, which are elongated on sagittal T2 WI (white arrow) **(E)**.

Supplementary Figure 6 Central nervous system MRI of case 4. Brain axial FLAIR **(A,B)** and the corresponding post-contrast T1 WI images **(C,D)** show tumefactive hipersignal intensity lesions with irregular and discontinued peripheral contrast enhancement (white arrow), determining mass effect predominantly in the frontal-parietal left lobe, such as in an ADEM pattern. Sagittal STIR and post-contrast T1 WI of the cervical (E,F) and dorsal **(G,H)** spinal cord have confluent and extensive hypersignal lesions (white arrows), with transversal damage in the dorsal area, resembling LETM pattern.

Supplementary Figure 7 Central nervous system MRI of case 5. Sagittal STIR of cervical **(A)** and dorsal (B) spinal cord with multiple elongated hypersignal images peripherally located along the white matter tracts (white arrows), associated with elongated hypersignal images in a radial distribution, from the central spinal cord gray matter to the peripheral white matter, best identified on axial T2 images **(C)**.

Supplementary Figure 8 Central nervous system MRI of case 6. Hyperintense lesion in the central pons identified on axial FLAIR **(A)** and T2-WI (white arrows) **(B)**, in the medium cerebellar peduncles. There is diffuse leptomeningeal gadolinium enhancement noticed on post-contrast axial FLAIR images (white arrow) **(D)** but non-observed on pre-contrast FLAIR **(C)**. Anterior horn diffuse damage is noticed on sagittal T2-WI of the cervical **(E)** and dorsal **(F)** spinal cord, which is confirmed on axial images (white arrow) **(G,H)**.

Supplementary Table 1 Clinical and neurological manifestations associated with virus diagnosis and systemic prodrome interval, cerebrospinal fluid results, comorbidities, and outcomes.

Supplementary Table 2 Pathogenic profile variants identified by whole-exome sequencing analysis.

Supplementary Table 3 Distribution of HLA alleles predicted from exomes of 6 patients with CHIKV-related CNS IDD and 6 patients with CHIKV without IDD.

Supplementary Table 4 Gene Ontology categories ranked for the 29 genes filtered in our analysis.
