# Supplementary figures and images for: Exome-Wide Search for Genes Associated With Central Nervous System Inflammatory Demyelinating Diseases Following CHIKV Infection: The Tip of the Iceberg
Source: Front Genet. 2021 Mar 17;12:639364. doi: 10.3389/fgene.2021.639364 (PMC8010313; doi:10.3389/fgene.2021.639364)

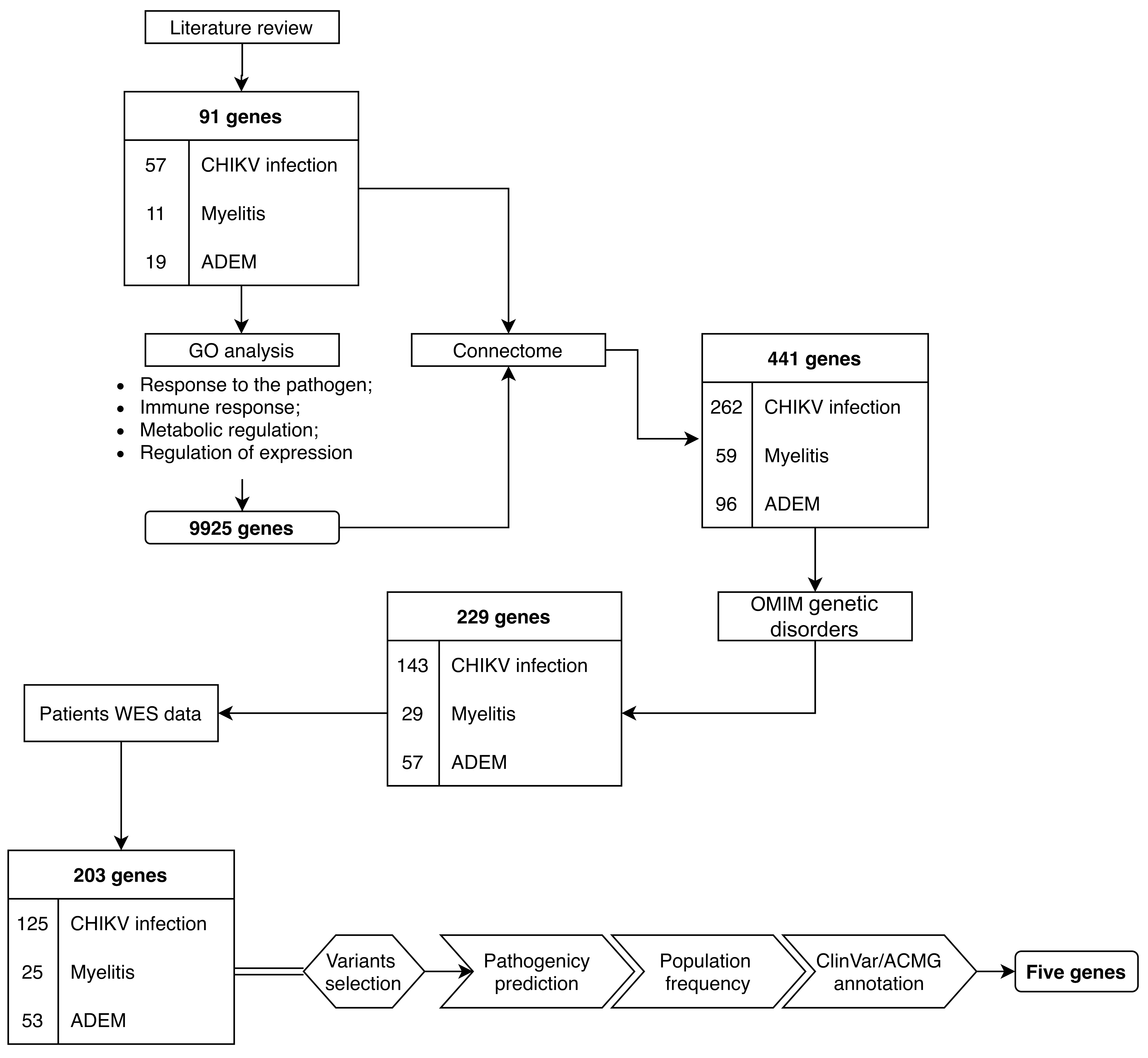

Supplement: Supplementary Figure 1 — Flowchart of gene panel prioritization. Strategies of variant filtration based on gene panel selection for CHIKV infection and neurological impairment. The main genes related to CHIKV infection, Myelitis and ADEM were firstly retrieved from the literature. Next, we expanded the gene set by retrieving genes related to enriched GO terms. To further investigate the relationship between the two sets (genes from the literature and recovered from GO) a pairwise protein-protein interaction (connectome) was performed. We retrieved the mendelian inheritance model for each gene by querying the OMIM database. Finally, we prioritize possibly damaging variants mapped in the final genes list for each patient. [file Image_1.JPEG]

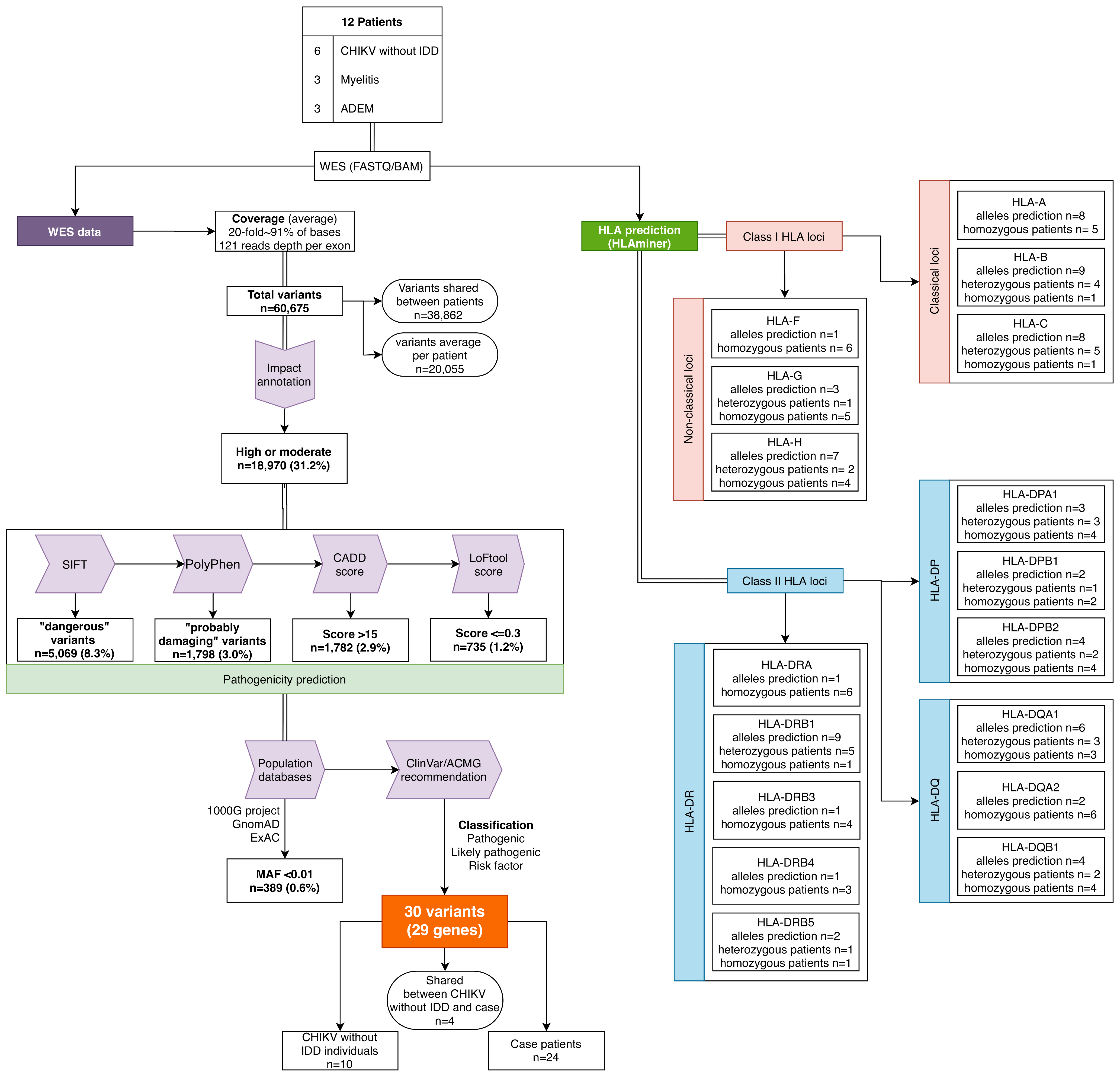

Supplement: Supplementary Figure 2 — Flowchart of the pipeline used to filter genetic variants and predict HLA alleles. From fastq files originated by WES, the pipeline was subdivided into two main approaches: (i) variant prioritization and (ii) HLA allele prediction. The set of variants selected was analyzed sequentially using computational tools to predict pathogenicity. They were then filtered by low frequency using population databases and further classified according to clinical guidelines from pathogenicity databases. Prediction of HLA alleles (class I and II) was performed with the HLAminer tool. [file Image_2.JPEG]

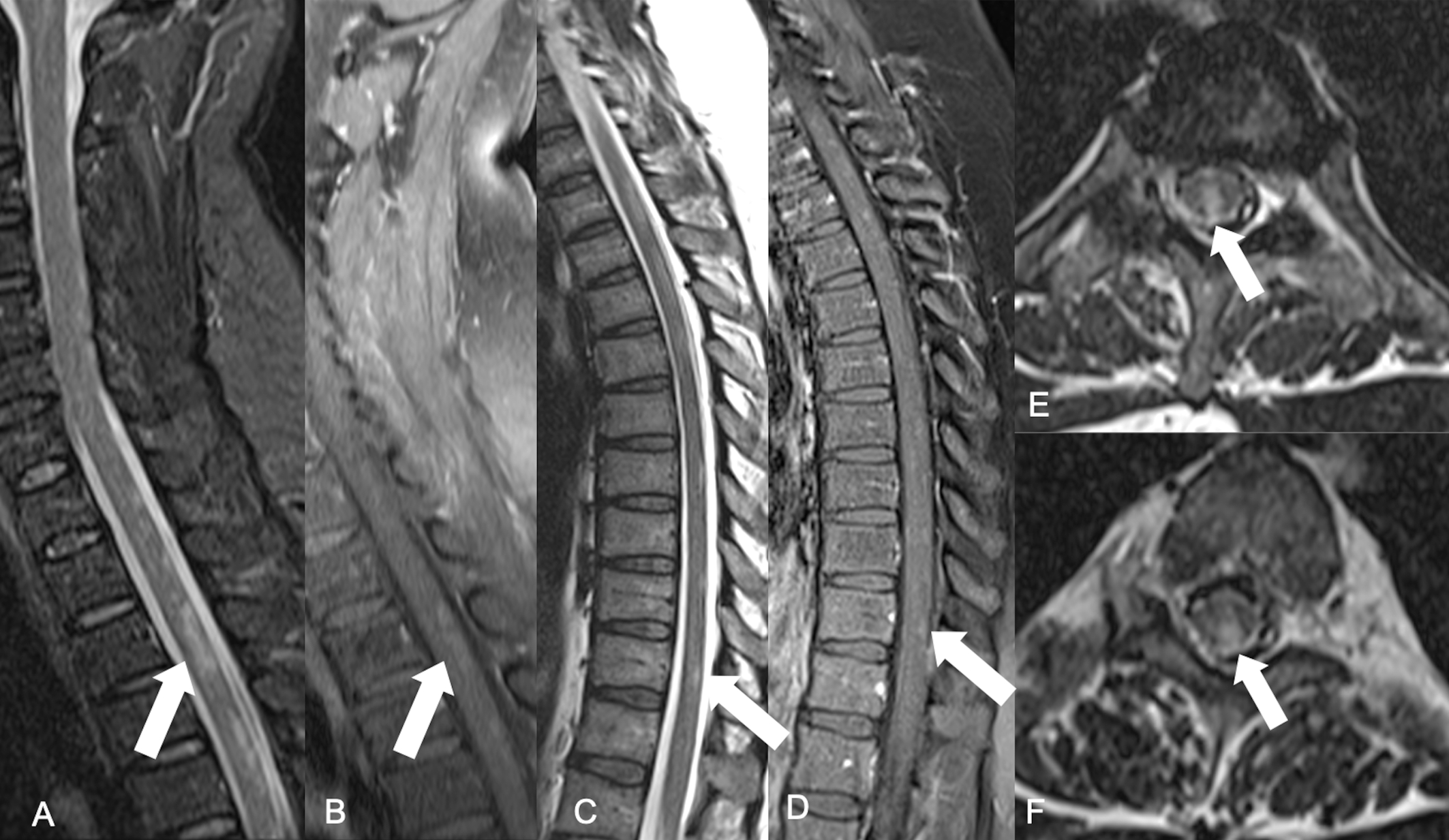

Supplement: Supplementary Figure 3 — Central nervous system MRI of case 1. Sagittal STIR (A) and T1 FS post-contrast (B) and sagittal T2 (C) and T1 FS post-contrast (D) of the cervical and dorsal spinal cord respectively show sparse hyperintense focci (white arrows), some with gadolinium enhancement, notably in the cervical-dorsal transition, extending <1 vertebral body, mostly affecting the posterior columns on axial T2 WI (white arrows) (E,F), resembling MS like pattern dissemination. [file Image_3.JPEG]

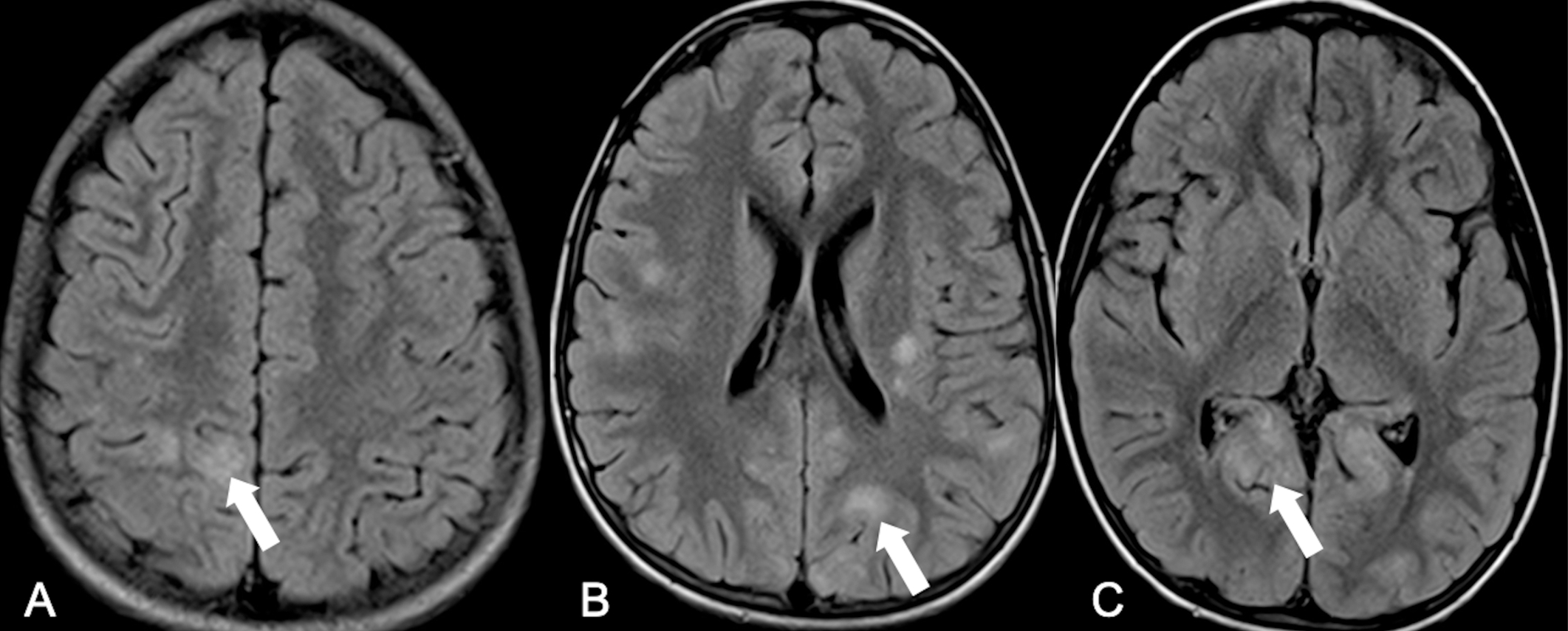

Supplement: Supplementary Figure 4 — Central nervous system MRI of case 2. Brain axial FLAIR images (A–C) show hyperintense cortical-justacortical lesions identified in the parietal and occipital lobes (white arrows), in the periventricular white matter and adjacent to the occipital horns of the lateral ventricles, characterizing an encephalitis pattern. [file Image_4.JPEG]

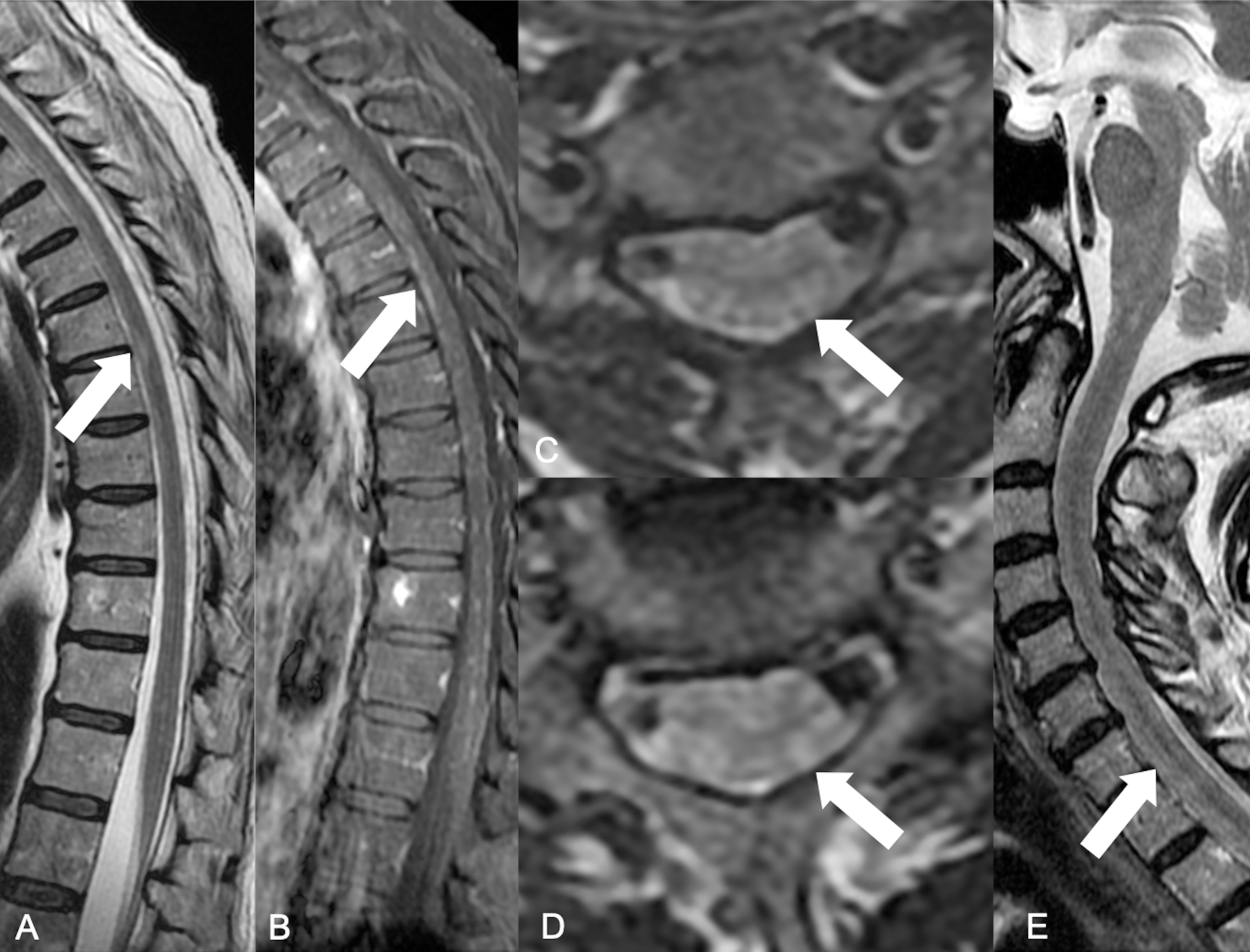

Supplement: Supplementary Figure 5 — Central nervous system MRI of case 3. Dorsal spinal cord sagittal T2 WI (A) have multiple peripheral hypersignal intensity focci (white arrows), with contrast enhancement on FS post-contrast T1 WI (B). Tiny focci of hypersignal intensity peripherally located on cervical axial T2 WI (C,D), which are elongated on sagittal T2 WI (white arrow) (E). [file Image_5.JPEG]

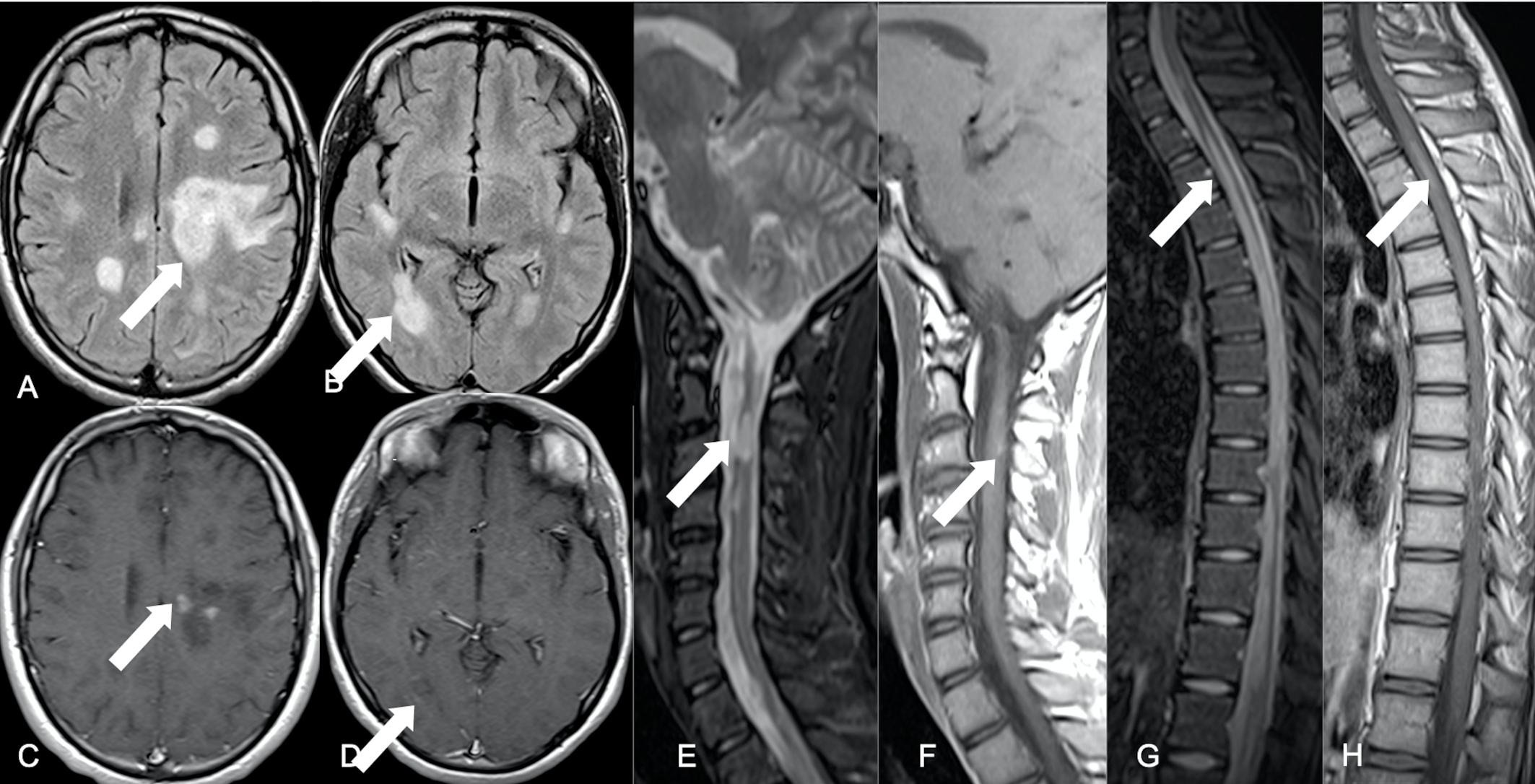

Supplement: Supplementary Figure 6 — Central nervous system MRI of case 4. Brain axial FLAIR (A,B) and the corresponding post-contrast T1 WI images (C,D) show tumefactive hipersignal intensity lesions with irregular and discontinued peripheral contrast enhancement (white arrow), determining mass effect predominantly in the frontal-parietal left lobe, such as in an ADEM pattern. Sagittal STIR and post-contrast T1 WI of the cervical (E,F) and dorsal (G,H) spinal cord have confluent and extensive hypersignal lesions (white arrows), with transversal damage in the dorsal area, resembling LETM pattern. [file Image_6.JPEG]

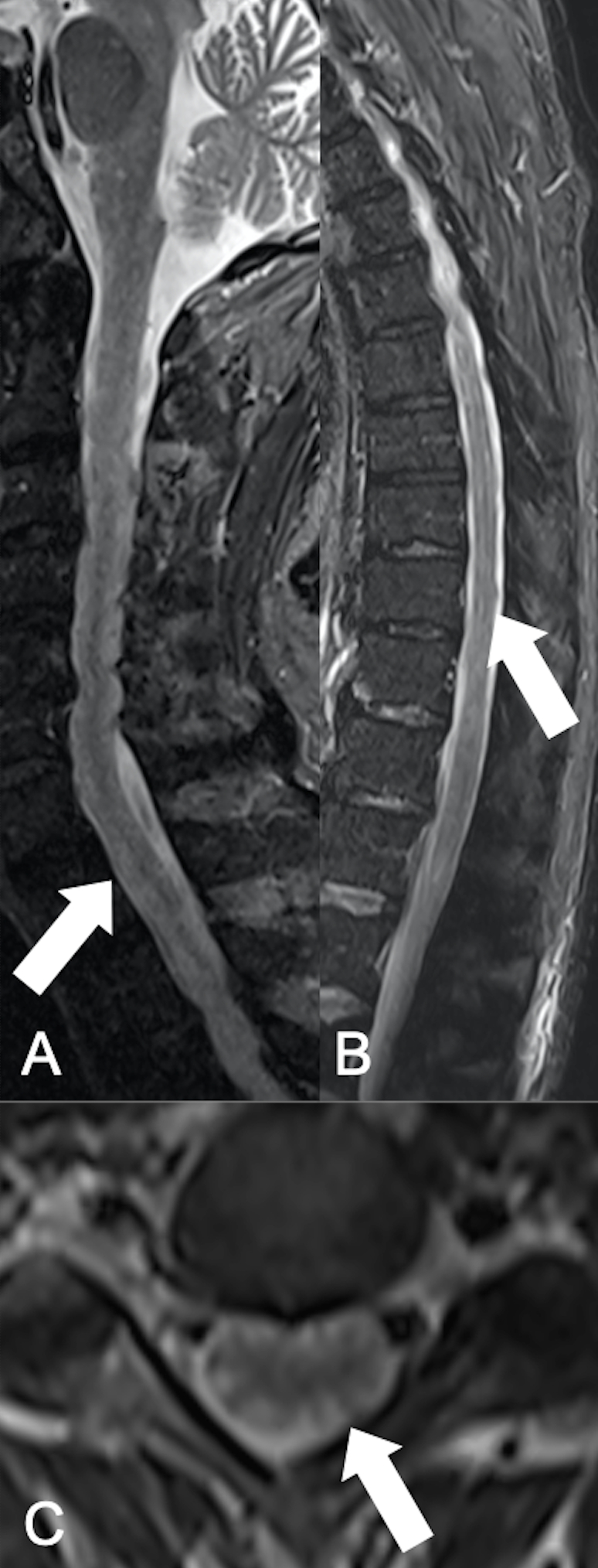

Supplement: Supplementary Figure 7 — Central nervous system MRI of case 5. Sagittal STIR of cervical (A) and dorsal (B) spinal cord with multiple elongated hypersignal images peripherally located along the white matter tracts (white arrows), associated with elongated hypersignal images in a radial distribution, from the central spinal cord gray matter to the peripheral white matter, best identified on axial T2 images (C). [file Image_7.JPEG]

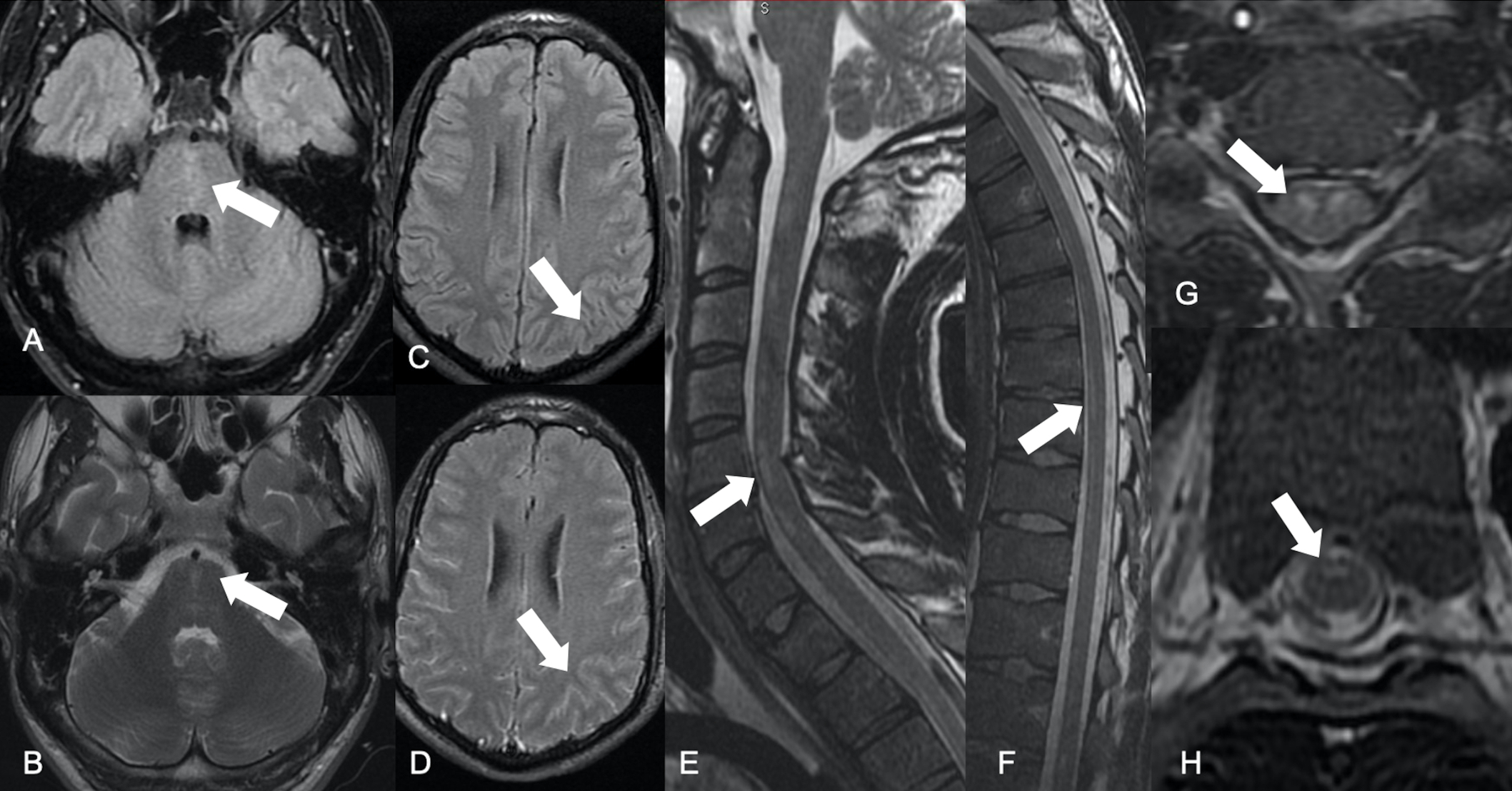

Supplement: Supplementary Figure 8 — Central nervous system MRI of case 6. Hyperintense lesion in the central pons identified on axial FLAIR (A) and T2-WI (white arrows) (B), in the medium cerebellar peduncles. There is diffuse leptomeningeal gadolinium enhancement noticed on post-contrast axial FLAIR images (white arrow) (D) but non-observed on pre-contrast FLAIR (C). Anterior horn diffuse damage is noticed on sagittal T2-WI of the cervical (E) and dorsal (F) spinal cord, which is confirmed on axial images (white arrow) (G,H). [file Image_8.JPEG]
